# Is Type 2 Diabetes a Primary Mitochondrial Disorder?

**DOI:** 10.3390/cells11101617

**Published:** 2022-05-12

**Authors:** Sarah Weksler-Zangen

**Affiliations:** 1 The Hadassah Diabetes Center, Hadassah Medical Center, Jerusalem 9112102, Israel; sarahz@hadassah.org.il; 2 The Liver Research Laboratory, Hadassah Medical Center, Jerusalem 9112102, Israel; 3 Faculty of Medicine Hebrew, University of Jerusalem, Jerusalem 9112102, Israel

**Keywords:** type 2 diabetes, mitochondria, cytochrome c oxidase, glucose stimulated insulin secretion, pancreatic β-cells, ATP

## Abstract

Diabetes mellitus is the most common endocrine disturbance in inherited mitochondrial diseases. It is essential to increase awareness of the correct diagnosis and treatment of diabetes in these patients and screen for the condition in family members, as diabetes might appear with distinctive clinical features, complications and at different ages of onset. The severity of mitochondrial-related diabetes is likely to manifest on a large scale of phenotypes depending on the location of the mutation and whether the number of affected mitochondria copies (heteroplasmy) reaches a critical threshold. Regarding diabetes treatment, the first-choice treatment for type 2 diabetes (T2D), metformin, is not recommended because of the risk of lactic acidosis. The preferred treatment for diabetes in patients with mitochondrial disorders is SGLT-2i and mitochondrial GLP-1-related substances. The tight relationship between mitochondrial dysfunction, reduced glucose-stimulated insulin secretion (GSIS), and diabetes development in human patients is acknowledged. However, despite the well-characterized role of mitochondria in GSIS, there is a relative lack of data in humans implicating mitochondrial dysfunction as a primary defect in T2D. Our recent studies have provided data supporting the significant role of the mitochondrial respiratory-chain enzyme, cytochrome c oxidase (COX), in regulating GSIS in a rodent model of T2D, the Cohen diabetic sensitive (CDs) rat. The nutritionally induced diabetic CDs rat demonstrates several features of mitochondrial diseases: markedly reduced COX activity in several tissues, increased reactive oxygen production, decreased ATP generation, and increased lactate dehydrogenase expression in islets. Moreover, our data demonstrate that reduced islet-COX activity precedes the onset of diabetes, suggesting that islet-COX deficiency is the primary defect causing diabetes in this model. This review examines the possibility of including T2D as a primary mitochondrial-related disease. Understanding the critical interdependence between diabetes and mitochondrial dysfunction, centering on the role of COX, may open novel avenues to diagnose and treat diabetes in patients with mitochondrial diseases and mitochondrial dysfunction in diabetic patients.

## 1. Diabetes and Pancreatic β-Cell Dysfunction

**Figure 1:**
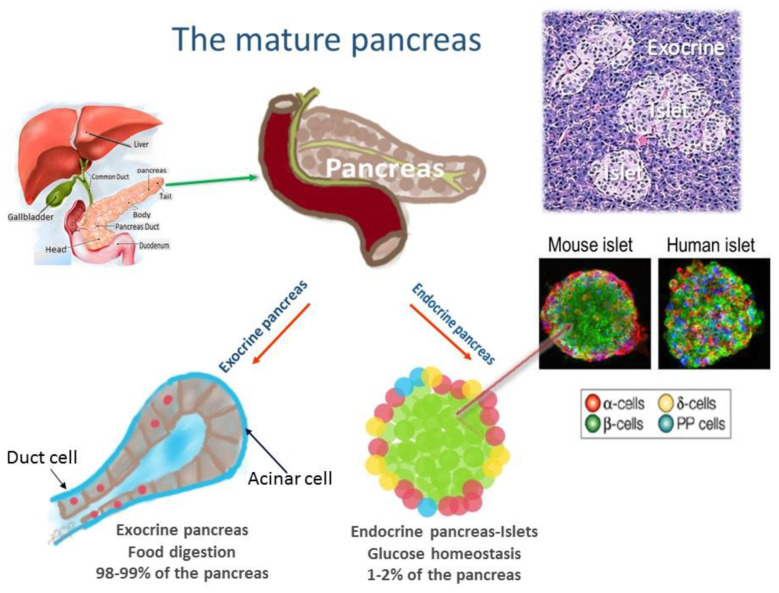
The pancreas is a mixed gland that contains both an endocrine and an exocrine digestive function. The pancreatic islets of Langerhans are irregularly shaped patches of endocrine tissue scattered within the pancreas. The islet includes five types of cells secreting different hormones: α cells produce glucagon released to the blood when glucose concentration is low (20% of total islet cells); β-cells, the only source of insulin in the body, release insulin when blood glucose concentration increases (≈55–70% of total islet cells); Δ cells produce somatostatin that inhibits insulin and glucagon secretion (<10% of total islet cells); ε cells produce ghrelin, the “hunger hormone” (<1% of total islet cells); PP cells or γ cells produce pancreatic polypeptide that regulates pancreatic secretion activities (<5% of total islet cells).

The absorption of blood glucose into the liver, fat, and skeletal muscle cells is dependent on insulin, a peptide hormone produced by β-cells in pancreatic islets (Figure 1). It is acknowledged that the actual manifestation of type 2 diabetes (T2D) occurs only when pancreatic β-cells fail to secrete sufficient insulin to stimulate glucose uptake in peripheral tissues. A combination of genetic and environmental factors results in progressive β-cell loss and dysfunction, leading to persistent hyperglycemia [1,2,3,4,5,6,7,8]. The estimated worldwide prevalence of people affected by T2D could reach 350 million by 2025 and rise [9]. Future individualized therapies for diabetes will require better characterization of the many pathways leading to β-cell dysfunction [7].

**Figure 2:**
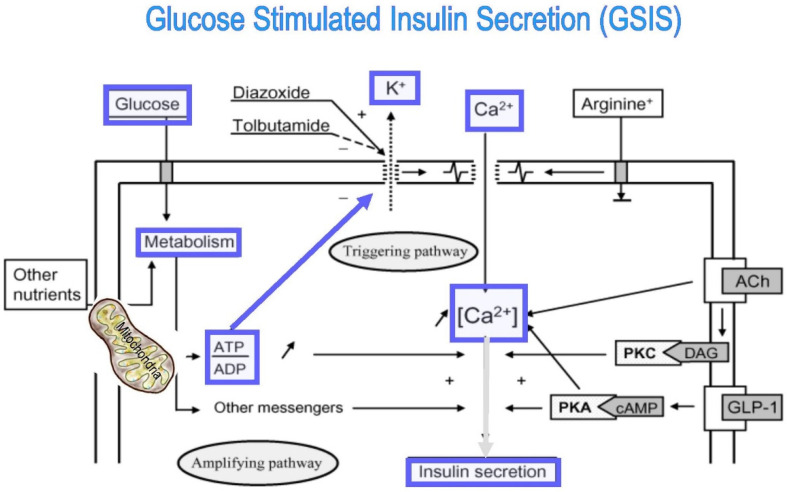
Glucose-stimulated insulin secretion (GSIS), the triggering pathway, includes entry of glucose into β-cells, acceleration of glucose metabolism, increase in ATP content and ATP/ADP ratio, closure of ATP-sensitive K^+^ channels (K^ATP^ channels), membrane depolarization, opening of voltage-dependent Ca^2+^ channels (VDCCs), increase in Ca^2+^ influx through VDCCs, raised intracellular Ca^2+^ concentration ([Ca^2+^] i), and exocytosis of insulin granules.

**Figure 3:**
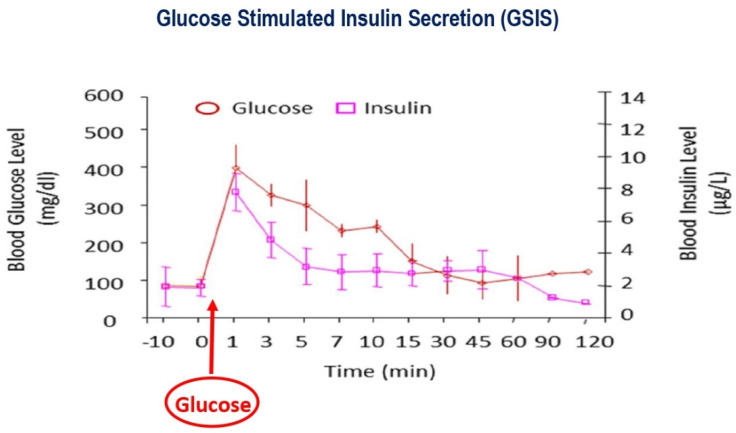
Glucose depends on insulin to enter the peripheral tissues, muscles, and fat. Without insulin, glucose stays in the blood, causing diabetes. The increase in blood glucose concentration triggers insulin secretion. Blood glucose levels, brown graph; the increase in blood glucose level triggers a prompt and parallel increase in insulin secretion, pink graph. With time, blood glucose levels reduce due to insulin’s facilitated entry into fat and muscles. In accord, reducing blood glucose levels is paralleled by lowering insulin levels.

Pancreatic β-cells are sensitive glucose sensors that adjust precise quantities of insulin release to variations in blood glucose levels critical to ensure adequate entry of glucose and homeostasis. The process of glucose-stimulated insulin secretion (GSIS) is exceptionally dependent on mitochondrial function (Figure 2 and Figure 3) [3,10,11,12,13,14,15,16,17].

## 2. Mitochondria and Insulin Secretion in β-Cells

**Figure 4:**
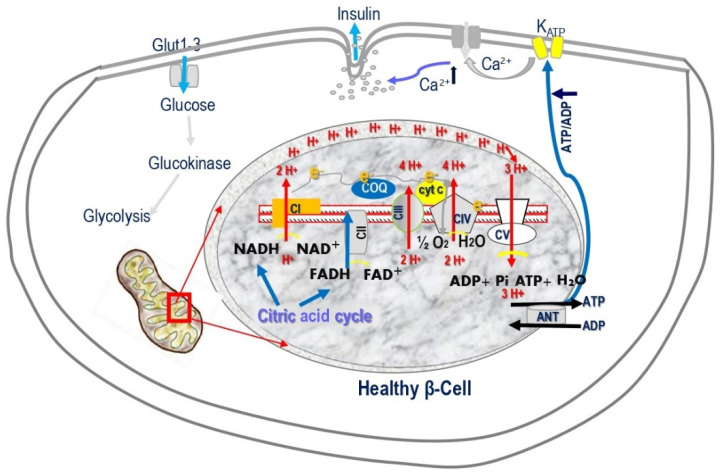
Mitochondrial oxidative phosphorylation (OXPHOS) is the only process that generates sufficient ATP to induce insulin secretion. OXPHOS is carried out by the mitochondrial respiration chain (MRC) located in the mitochondrial inner membrane. Electrons move between the four complexes and are transferred to oxygen by complex IV, cytochrome coxidase (COX). The free energy released pumps protons at complexes I, III, and IV to the intermembrane space, creating a proton gradient. At complex V, ATP synthase, protons diffuse along this electrochemical gradient to generate ATP.

ATP production in β-cells is crucial for stimulus-secretion coupling, a cascade of molecular events encompassing the initial sensing and transport of glucose to β-cells, triggering the exocytosis of insulin. GSIS requires increased mitochondrial ATP generation up to the metabolic threshold required for robust insulin release [3,10,11,12,13,14,15,16,17]. Mitochondrial oxidative phosphorylation (OXPHOS) is the only process that couples glucose metabolism and generates sufficient ATP required to close K^ATP^ channels and stimulate insulin secretion (Figure 2 and Figure 4). Glucose elevation triggering OXPHOS is carried out by five mitochondrial respiratory-chain (MRC) multimeric-enzyme complexes, utilizing reduced coenzymes, reduced nicotinamide adenine dinucleotide (NADH), and reduced flavin adenine dinucleotide (FADH_2_) [12,15,16,17]. These four MRC complexes are located in the mitochondrial inner membrane, and coenzyme Q and cytochrome c are located in the inner membrane space. Complexes I, III, and IV (cytochrome c oxidase, COX) utilize the energy from the electron transfer to pump protons from the matrix across to the intermembrane space, creating an electrochemical gradient used to generate ATP via complex V (ATP synthase). COX catalyzes the last rate-limiting electron transport chain (ETC) step, receiving electrons from cytochrome c and transferring them to oxygen (Figure 2 and Figure 4) [11,18,19,20]. Impairment of β-cell mitochondrial ATP production leads to insufficient insulin release [2,3,5,10,11,12,13,14,15,16,21,22,23,24,25,26,27,28,29,30,31]. β-cells develop special features to ensure a tight coupling of glucose concentration to insulin secretion. β-cells perform cell-specific gene expression and repression of specific housekeeping genes. They express high metabolic sensing enzymes, including the glucose transporter GLUT2 and glucokinase, and selectively suppress enzymes such as lactate dehydrogenase A (LDHA) and the pyruvate/lactate transporter monocarboxylic acid transporter (MCT-1), which are considered genes “disallowed” to shuttle glucose, via pyruvate, to the mitochondria to fuel ATP production [6,32,33]. To replenish the cytosolic NAD^+^ and FADH electron donors, β-cells acquire unique shuttles, the malate–aspartate shuttle and the glycerophosphate shuttle [13,34].

**Figure 5:**
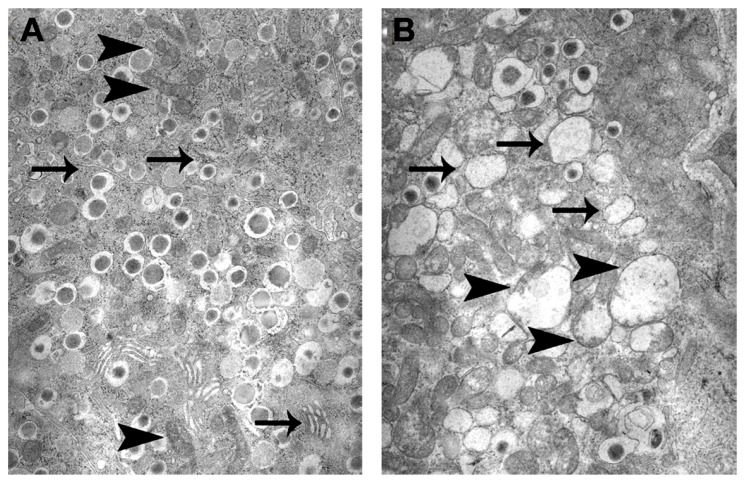
Photomicrographs of pancreas sections of hyperglycemic Cohen diabetic rats [8,10,47]. (**A**) The β-cells in the center of the islets exhibited well-preserved mitochondria (arrow) and normal ER (arrowhead). The β-cells in the periphery of the islets (**B**) showed swollen mitochondria (arrowhead) and dilated ER. EM magnification 18,000×.

Mitochondrial dysfunction in T2D: Despite the well-characterized role of mitochondria in β-cell stimulus-secretion coupling, there is a relative scarcity of data in humans implicating mitochondrial dysfunction in the pathogenesis of T2D. Observation in T2D patients supports the link between reduced ATP generation by the mitochondria and β-cell dysfunction. Abnormal mitochondrial morphology and reduced GSIS are found in β-cells from postmortem T2D patients [15,35,36,37,38,39]. Round and swollen mitochondria impact mitochondrial function, driving impaired GSIS, as shown in a study in which islets from T2D subjects failed to reverse hyperglycemia when transplanted into diabetic mice, while islets from healthy humans in equivalent numbers managed to do so [37]. T2D islets had a significantly lower selective response to glucose, whereas the response to non-nutrient secretagogues arginine and glipalamides remained, a phenomenon characteristic of β-cells with mitochondrial dysfunction [2,8,11,12,17]. Patients with mitochondrial diabetes harbor point mutations or deletions in mtDNA [40,41,42,43]. Swollen, larger mitochondria also appear in mice fed a high-fat diet for 12 weeks [44]. In our studies, we found reduced GSIS and hyperglycemia in tight association with swollen, larger mitochondria and endoplasmic reticulum (ER) in islets of the Cohen diabetic sensitive (CDs) rat, a unique, inbred, nutritionally induced T2D rat model (Figure 5A,B) [8,45,46,47]. CDs rats maintain normoglycemia on a regular diet (RD) but develop hyperglycemia after exposure to a diabetogenic diet related to markedly reduced GSIS [8,46,47,48]. Our recent observations demonstrated a significant correlation (*p* < 0.01) between CDs islet-COX activity and GSIS, implicating a critical role of islet-COX activity as a modulator of GSIS [10].

**Figure 6:**
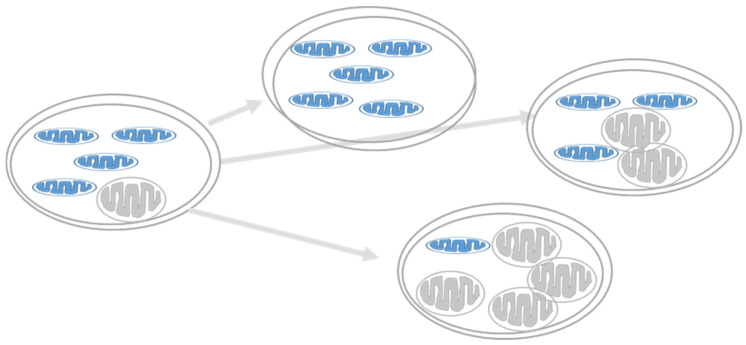
Heteroplasmy and diabetes. Heteropalsmy is the relative proportion of mtDNA copies carrying the mutation in a specific tissue. A specific phenotype develops when a certain threshold level of mutation is reached (usually ~60%) [55].

Human mitochondrial DNA (mtDNA) is a circular, double-stranded, supercoiled molecule that encodes 37 genes, essential for OXPHOS and mitochondrial protein synthesis [49]. There are approximately 1000 mitochondrial genomes in most cells, and their replication is independent of the cell cycle. mtDNA has a 10–20 times higher replication rate than nuclear DNA (ntDNA), which can explain the much higher rate of sporadic mutations in mtDNA. It is common for mutations to affect only some of the hundreds of mitochondria copies in the cell, leaving many unaffected. Heteroplasmy is the presence of more than one type of mitochondrial DNA within one cell (Figure 6). A biochemical threshold is associated with an increased mutant mtDNA percentage causing decreased OXPHOS function and phenotype onset, as described in classical mitochondrial diseases explaining the different phenotypes for comparable mutations and the other ages of onset, as well as the severity of the disorder [50,51]. In β-cells, the tight coupling between glucose metabolism and insulin secretion is exclusively dependent on the mitochondria to generate sufficient ATP production required for insulin secretion. This is a pivotal situation in β-cells because increasing mitochondrial function ensues reactive oxygen species (ROS) production, which has a deleterious effect on its function. β-cells are highly sensitive to oxidative stress due to their low antioxidant defense mechanism, the lack of protective histones, and poor DNA-repair mechanisms [52,53,54]. Thus, the accumulation of mtDNA mutations in β-cells could increase the prevalence of mitochondrial-related reduced GSIS and T2D [14,50].

## 3. Organization of The Electron Transport Chain

Mitochondria function relies on their structure and the organization of OXPHOS complexes. MRC complexes are arranged in the inner mitochondrial membrane as a “respirasome” [56,57]. The respirasome are supercomplexes made of complexes I, III, and IV. This arrangement allows substrate channeling or direct transfer of electrons from one enzyme to another, providing kinetic advantage, efficient electron flow, and stability, thereby reducing electron loss and ROS generation [3,56]. In accord, fused or fragmented mitochondria observed in islets of T2D donors exhibited selective impairment in GSIS [35,58,59,60,61]. Defective assembly of MRC supercomplexes was associated with diabetes in animal models [62] and the rectus abdominis muscle of obese individuals with T2D [63]. Reduced expression of a set of OXPHOS genes affecting the generation of MRC complex subunits was observed in islets of T2D patients and animal models of diabetes [64,65,66]. These observations further support a critical role of mitochondrial dysfunction and subsequent ROS overproduction in the pathogenic process leading to β-cell dysfunction. Yet, the existence and functional significance of respirasomes in islet β-cells are still under investigation.

## 4. Cytochrome Coxidase (COX)

**Figure 7:**
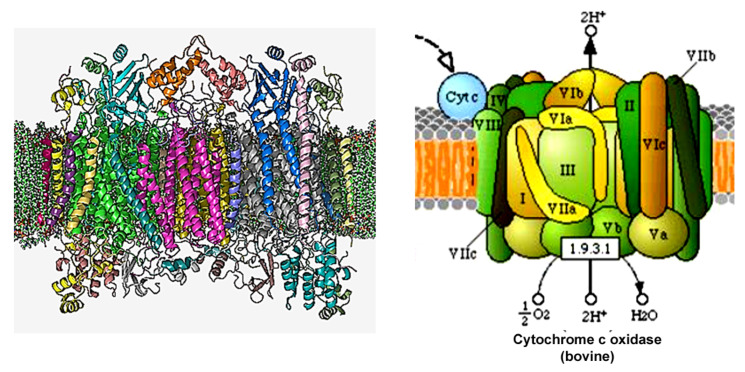
(**Left**) The crystal structure of bovine homodimer cytochrome c oxidase in a phospholipid bilayer. Adapted from The Protein Data Bank (PDB) PDB: 1OCC. PDB structures: RCSB PDB PDBe PDBsum. Diagrammatic representation of COX's subunits in different colors. (**Right**) A diagram of COX taken from the KEGG pathways (https://www.genome.jp/kegg-bin/show_pathway?map00190 accessed on 5 February 2022).

COX plays a vital role in regulating mitochondrial respiration and OXPHOS to adjust ATP production to specific-cellular energy requirements, depending on nutritional or environmental stimuli. However, COX activity in pancreatic β-cells probably differs from other cell types, as it requires firm and tight regulation to ensure that the rate of insulin secretion closely mirrors the change in blood glucose levels. β-cells are totally dependent on OXPHOS for ATP production and cannot accelerate glycolysis to compensate for decreased OXPHOS due to repressed lactate dehydrogenase expression. COX adapts to changing environmental conditions [19,20]. Proper assembly of COX catalytic subunits is essential for its function. Any change or removal of nuclear subunits results in impaired COX activity and impairs the formation of supercomplexes, while mutations cause different mitochondrial diseases [67,68] (Figure 7). However, the role of COX-impairment in human T2D is not established. COX catalyzes the ETC rate-limiting step of OXPHOS, receiving electrons from cytochrome c and transferring them to oxygen. The three mitochondrial encoded COX subunits, subunits 1, 2, and 3 (COX1, COX2, COX3), build the catalytic core of the enzyme. There is a debate regarding the number of nuclear-encoded subunits that regulate, stabilize, assemble, and anchor the enzyme complex to the inner membrane of the mitochondria [19,67,68,69]. Originally, 13 subunits were reported, but recently, NDUFA4 was identified as a subunit of complex I and was suggested to be the 14th subunit of COX [19,70,71,72]. COX is usually found as a dimer, but monomeric crystal structures have also been reported to form supercomplexes, suggesting that equilibrium could exist between the dimeric and monomeric forms of COX [19,70,72]. NDUFA4 was meant to bind to the monomeric form of COX, thereby excluding the formation of COX dimers [19]. Under this proposal, NDUFA4 could bind complex I or COX under specific situations. Kadenbach et al. suggest two forms of COX: a “relaxed state” and an “active state”. The relaxed state maintains adequate membrane potential and prevents ROS generation. In this state, allosteric ATP inhibition of the phosphorylated and dimeric COX maintains a low and acceptable mitochondrial membrane. In the “active state”, dephosphorylated monomeric COX binds to NDUFA4, stabilizing it in its monomeric form. In stress situations, allosteric ATP inhibition is abolished by Ca^2+^-activated dephosphorylation, leading to monomerization and NDUFA4 moving from complex I to COX. The “active state” induces higher rates of COX activity and ATP synthesis but increases ROS formation, decreasing mitochondrial efficiency [19,73]. Excessively produced ROS in mitochondria participate in the generation of multiple diseases, including diabetes mellitus and mitochondrial disorders [11,16,18,52,68,74]. In a recent study, decreased COX activity and increased ROS production were observed in streptozotocin-diabetes-induced rats [74], and another study showed that in the diabetic state, COX activity was decreased in islets [64]. In a recent study, we demonstrated that COX activity was initially ~30% reduced in islets of normoglycemic CDs. When fed a diabetogenic diet, islet-COX activity reduced to ≥46% of baseline, resulting in a parallel decrease in GSIS and increase in blood glucose levels. The progressive reduction in COX activity in CDs islets positively correlated with decreasing GSIS (R^2^ = 0.9691, *p* < 0.001) and inversely with the elevation in blood glucose levels (R^2^ = 0.8396, *p* < 0.001), supporting the notion that islet-COX activity could be a major metabolic regulator of β-cell function [10]. Moreover, our study suggests that reduced islet-COX activity could be a primary inborn defect that underlies the decrease in β-cell dysfunction when exposed to environmental stressors [10]

## 5. Which Comes First? Diabetes or Mitochondrial Dysfunction?

**Figure 8:**
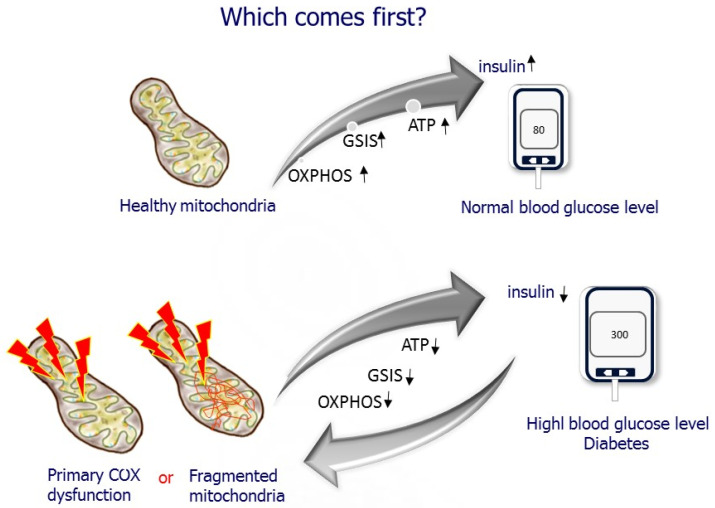
Which comes first? Does mitochondrial dysfunction cause diabetes, or does diabetes hamper mitochondrial function?

The presence of β-cell dysfunction as an early defining event in the onset and progress of T2D is currently the predominantly accepted view [13,15,22]. However, the cellular and molecular mechanism underlying β-cell dysfunction and what is the major contributor are unresolved. The question is, which comes first? Does hyperglycemia cause deleterious effects on β-cell mitochondria? In support of this assumption, studies have demonstrated changes in β-cell metabolism, and markedly reduced mitochondrial function has been observed in isolated islets from T2D donors, animal models of diabetes, human islets, and β-cell lines exposed to high glucose levels [22,35,75,76,77,78]. On the other hand, defective mitochondrial function decreases GSIS, as demonstrated in studies using drugs affecting the MRC and mutations and depletion of the mitochondrial genome [3,12,13,15]. However, most of those studies relate ETC dysfunction to excessive ROS generation causing cellular injury. β-cell overstimulation has recently been proposed as the starting point initiating the harmful process of β-cell dysfunction in the face of insulin resistance [13,22]. Our studies suggest the possibility of reduced islet-COX activity as a primary cause of β-cell dysfunction in T2D in a rat model that does not exhibit insulin resistance centering islet-COX dysfunction [8,10,46,47], as described in the following sections (Figure 8).

## 6. COX Activity as a Primary Cause of β-Cell Dysfunction in T2D

**Figure 9:**
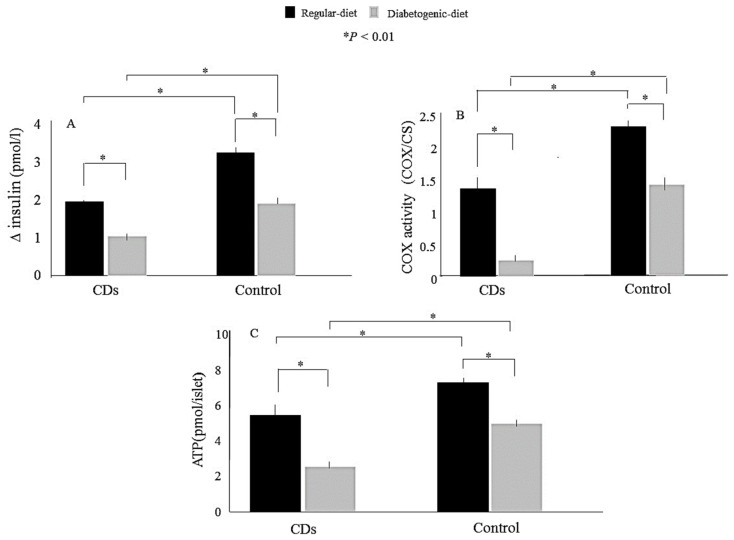
(**A**) Glucose-stimulated insulin secretion (GSIS), (**B**) cytochrome c oxidase (COX) activity, and ATP content (**C**) in islets isolated from Cohen diabetic sensitive (CDs) rats or control rats fed either a regular diet (black bars) or a diabetogenic diet (gray bars). (**A**) GSIS measured at 1.7 (baseline) and 16.7 mmol/L (stimulatory) glucose concentrations. (**B**) COX activity normalized to citrate synthase (CS) activity (COX/CS). (**C**) ATP content (pmol/islet) determined by luciferin luciferase. Data are means ± SE for at least three separate experiments. * *p* < 0.01. Figure is used and modified from [45] with permission from the publisher.

We examine whether mitochondrial dysfunction could precede hyperglycemia promoting diabetes development in an inbred, lean model of nutritionally induced diabetes, the Cohen diabetic sensitive (CDs) rat [8,47]. The CDs rat develops hyperglycemia only when fed a diabetogenic diet but maintains normoglycemia on a regular diet (RD). CDs rats do not exhibit insulin resistance and hyperglycemia due to selective impairment in GSIS. Insulin secretion induced by non-nutrient secretagogues arginine and tolbutamide is sustained, suggesting the selective GSIS impairment related to mitochondrial deficiency. Moreover, diabetes is nutritionally induced in this model, providing the opportunity to study the relationship between GSIS and COX activity at the prediabetic state, at different stages during diabetes development, and at a full diabetic condition [8,46,47]. We found that COX activity was initially reduced by 50% in the CDs rats before diabetes development. Islets isolated from hyperglycemic CDs rats exhibited markedly diminished COX activity, leaving only 15% residual activity, thereby inducing a comparable decrease in ATP production and GSIS (Figure 9) [45]

**Figure 10:**
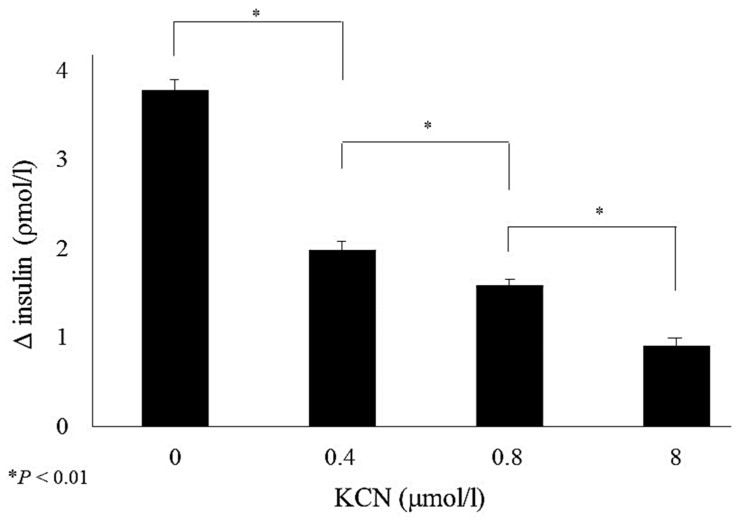
Glucose-stimulated insulin secretion of islets isolated from control rats fed a regular diet and incubated for 1 h with either 0, 0.4, 0.8, or 8 mol/L potassium cyanide (KCN) in the presence of 1.7 (baseline) or 16.7 mmol/L (stimulatory) glucose. The figure expresses insulin secretions measured as insulin secreted under stimulatory glucose minus insulin secreted under baseline glucose concentrations per islet of insulin content. Data are means ± SE for at least three separate experiments, * *p* < 0.01. Figure is used from [45] with permission from the publisher.

We also demonstrate a direct link between inhibition of COX activity and insulin secretion by incubating isolated islets with the COX-specific inhibitor, potassium cyanide, and demonstrating an apparent dose-dependent reduction in insulin secretion in isolated islets exposed to different doses of potassium cyanide (Figure 10).

Interestingly, we also found that reduced islet-COX activity and GSIS were highly associated with the pancreatic inflammatory process featured by peri-islet infiltration of fat and activated macrophages releasing the proinflammatory cytokine interleukin 1-beta (IL1-β). IL1-β dose-dependently diminishes GSIS by substantially increasing nitrite levels [8,45]. The increased NO production may interact and inhibit the function of several mitochondrial respiratory-chain components by reversible S-nitrosation. However, our data support the notion that NO competes with the oxygen binding, inhibiting COX. Moreover, the interference with ETC favors the production of reactive oxygen species, which by further interaction with nitric oxide (NO) form peroxynitrite, inducing cellular damage [53,79,80,81]. Hyperglycemia was fully counteracted by treating the CDs rats with specific-IL-1β antibodies [82].

**Figure 11:**
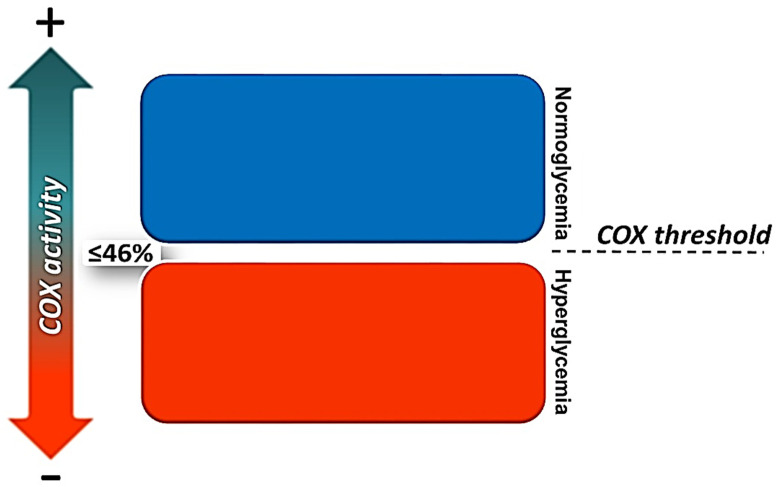
The islet-COX activity threshold. In CDs rats, islet-COX activity of ≥46% is mandatory to maintain normoglycemia [10].

In a recent study, we linked reduced mitochondrial COX activity in islets, insulin secretion, and glucose homeostasis. We demonstrated that hyperglycemia was initiated when islet-COX activity decreased below 46% of baseline, identifying a novel islet-COX activity threshold required to sustain normoglycemia (Figure 11). We demonstrated a progressive reduction in COX activity in CDs islets that correlated positively with the decreasing GSIS (R^2^ = 0.9691, *p* < 0.001) and inversely with the elevation in blood glucose levels (R^2^ = 0.8396, *p* < 0.001), suggesting that islet-COX activity could be a significant metabolic sensor in pancreatic β-cells. Our studies concluded that: (1) islet-COX activity is directly associated with GSIS; (2) pancreatic inflammation reduced GSIS via NO production triggered by IL-1β secreted from peri-islet-infiltrating macrophages; (3) COX activity was markedly reduced in hyperglycemic CDs due to a pre-existing inborn (primary) impairment in mitochondrial respiratory chain dysfunction setting the initial islet-COX activity at ≤46% of baseline, thus enabling an almost complete inhibition of islet-COX activity, diminished GSIS and increased blood glucose levels.

## 7. Is Diabetes a Mitochondrial Disease? What Is the Therapeutic Importance?

Diabetes mellitus is not a classical mitochondrial disease (Table 1). T2D lacks typical features of encephalopathy, lactic acidosis, and stroke-like episodes. Yet, it is the most common endocrine disease in inherited mitochondrial diseases [83,84]. So far, only variations in essential OXPHOS genes relate to mitochondrial dysfunction and reducing insulin secretion were reported in T2D [65,83,84,85]. Pancreatic β-cells maintain glucose homeostasis by “coupling”, fine-tuning glucose metabolism to insulin secretion, a process that requires OXPHOS. In accord, β-cells have unique features to ensure ATP generation by OXPHOS and adequate GSIS, including suppression of housekeeping genes (“disallowed” genes) that are usually expressed in other cells [6,32,33,86]. Yet, despite the well-characterized role of mitochondria in β-cell stimulus-secretion coupling, there is a relative lack of data in humans implicating mitochondrial dysfunction as a primary defect in T2D. Our studies provide evidence implicating reduced islet-COX activity as a primary event in the decreasing GSIS in pancreatic islets of CDs rats [10,45,46].

In conclusion, T2D is highly dependent on the mitochondria and as such could be considered a mitochondrial-associated disease. However, the question of whether it is a primary mitochondrial disease remains open. Diabetes mellitus is part of the pathologies of the mitochondrial disease exhibiting distinctive clinical features and complications that are not found in classical diabetes [41,84]. It is therefore essential to identify these patients and provide the correct treatment. Metformin, the first-choice treatment in T2D is not recommended because of the risk of lactic acidosis [87], while SGLT-2i and GLP-1-related substances improving mitochondrial functions have significant benefits for diabetic patients with identified mitochondrial mutations [88].

## Figures and Tables

**Table 1:** T2D as mitochondrial disease.

Pros	Cons
mtDNA mutations or deletion of mitochondrial genes reduce GSIS capacity and causes diabetes	Most mtDNA mutations found were not causatively related to diabetes
Deficient mitochondrial function are found in many diabetes patients	Mitochondrial dysfunction might be caused by hyperglycemia
Mitochondrial dysfunction or deletion reduced or inhibit GSIS	No sufficient clinical data to implicate mitochondrial dysfunction as a primary cause of T2D
During the development of overt T2D, mitochondria appear round and swollen	Mitochondrial structure abnormalities could be ensued by diabetes
Comparable to mitochondrial diseases, GSIS is markedly reduced but insulin secretion in response non nutrient secretagogue sustain	No clinical manifestation of classical mitochondrial disease such as encephalopathy lactic acidosis & stroke like episodes
ATP generation up to the metabolic threshold for robust insulin secretion release requires ATP generation by OXPHOS	
β-cells have unique features to ensure ATP generation by OXPHOS and adequate GSIS such as suppression of housekeeping genes that are normally expressed in other cells	
Islets of T2D patients exhibit a variation in the expression of OXPHOS and metabolic genes that are likely to reduce GSIS and cause diabetes	
Variants in the mitochondrial transcription factor TFB1M have been implicated by GWAS of T2D	
Loss of the mitochondrial protein frataxin impairs the activity of the electron transport chain (ETC), leading to a higher incidence of diabetes in Friedreich ataxia patients	

## Data Availability

Not applicable.
